# Understanding mosquito host-choice behaviour: a new and low-cost method of identifying the sex of human hosts from mosquito blood meals

**DOI:** 10.1186/s13071-021-04577-w

**Published:** 2021-01-22

**Authors:** Fiona Teltscher, Sophie Bouvaine, Gabriella Gibson, Paul Dyer, Jennifer Guest, Stephen Young, Richard J. Hopkins

**Affiliations:** 1 Natural Resources Institute, University of Greenwich, Central Avenue, Chatham Maritime, Kent, ME4 4TB UK; 2 Anglia Ruskin University, East Road, Cambridge, CB1 1PT UK; 3 Home Office, Lunar House, 40 Wellesley Road, Croydon, CR9 2BY UK

**Keywords:** Mosquitoes, Vector-borne diseases, Host choice, Blood-feeding behaviour, Epidemiology

## Abstract

**Background:**

Mosquito-borne diseases are a global health problem, causing hundreds of thousands of deaths per year. Pathogens are transmitted by mosquitoes feeding on the blood of an infected host and then feeding on a new host. Monitoring mosquito host-choice behaviour can help in many aspects of vector-borne disease control. Currently, it is possible to determine the host species and an individual human host from the blood meal of a mosquito by using genotyping to match the blood profile of local inhabitants. Epidemiological models generally assume that mosquito biting behaviour is random; however, numerous studies have shown that certain characteristics, e.g. genetic makeup and skin microbiota, make some individuals more attractive to mosquitoes than others. Analysing blood meals and illuminating host-choice behaviour will help re-evaluate and optimise disease transmission models.

**Methods:**

We describe a new blood meal assay that identifies the sex of the person that a mosquito has bitten. The amelogenin locus (*AMEL*), a sex marker located on both X and Y chromosomes, was amplified by polymerase chain reaction in DNA extracted from blood-fed *Aedes aegypti* and *Anopheles coluzzii*.

**Results:**

*AMEL* could be successfully amplified up to 24 h after a blood meal in 100% of *An. coluzzii* and 96.6% of *Ae. aegypti*, revealing the sex of humans that were fed on by individual mosquitoes.

**Conclusions:**

The method described here, developed using mosquitoes fed on volunteers, can be applied to field-caught mosquitoes to determine the host species and the biological sex of human hosts on which they have blood fed. Two important vector species were tested successfully in our laboratory experiments, demonstrating the potential of this technique to improve epidemiological models of vector-borne diseases. This viable and low-cost approach has the capacity to improve our understanding of vector-borne disease transmission, specifically gender differences in exposure and attractiveness to mosquitoes. The data gathered from field studies using our method can be used to shape new transmission models and aid in the implementation of more effective and targeted vector control strategies by enabling a better understanding of the drivers of vector-host interactions. 
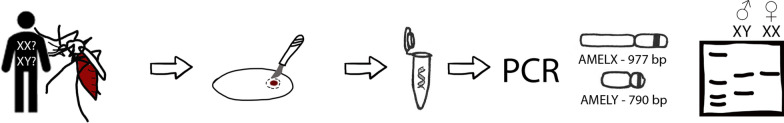

## Background

Mosquito-borne diseases are a global health problem, causing hundreds of thousands of deaths each year [1–3]. Malaria alone resulted in the death of at least 405,000 people in 2018, most of whom were children under 5 years of age [1]. Annually, there are between 67.1 and 135.6 million symptomatic cases of dengue, a viral disease transmitted by *Aedes* mosquitoes, which place an additional burden on already struggling health systems [4]. Recent outbreaks of Zika virus, also transmitted by *Aedes* mosquitoes, have been linked to an increase in the numbers of children born with microcephaly or other birth defects [5]. Mosquitoes need to take a blood meal, or probe, at least twice to transmit pathogens, making their blood-feeding behaviour of interest in epidemiological research. Two mosquito groups are of special importance in the transmission of vector-borne diseases. Certain *Anopheles* spp. are the most important vectors of malaria and are highly anthropophilic [6]. *Aedes aegypti* and *Aedes albopictus* are the most common vectors of dengue, and can also transmit a range of other diseases, including West Nile fever, Chikungunya and Zika [7]. In particular, dengue has been on the rise in recent years and is predicted to continue to spread due to climate change [8]. Better control strategies are required for all medically important mosquito vector species, as insecticide resistance is increasing in both *Ae. aegypti* [9] and *Anopheles* spp. [10]. In addition, it is likely that other vector-borne zoonotic diseases, especially arboviruses, will become an increasing problem due to mankind’s influence on the planet, e.g. climate change or changes in land use. Consequently, monitoring mosquito biting behaviour is vital to improve detection and prediction of the spread of vector-borne diseases [11].

Currently, it is possible to determine the host species on which a mosquito has fed, which helps researchers predict the possible presence of zoonoses, either through immunological methods, such as enzyme-linked immunosorbent assay (ELISA) [12, 13], or molecular methods, such as DNA sequencing [14–21], or the analysis of specific markers for further characterisation of arthropod blood meals [22]. Furthermore, the identity of individual human hosts from mosquito blood meals can be matched to specific inhabitants of nearby dwellings with short tandem repeat (STR) genotyping. This approach has shed light on mosquito feeding behaviour and has improved the success of monitoring vector control methods in malaria endemic regions in South India [23] and Kenya [24], and in areas at risk of dengue transmission [25, 26]. STR genotyping is expensive and requires the participation of local people to provide DNA samples for comparison with human DNA found in mosquitoes [23]. Ethical implications of collecting genetic material can arise [27], and it is not always possible to get samples from everyone who is at risk of being bitten in the sampling area; however, STR genotyping kits are still useful for determining the sex of a person who has been bitten, even though they cannot match blood-fed mosquitoes to a known donor.

There are three main factors underlying potential sex differences in the epidemiology of mosquito-borne diseases. Firstly, there is evidence that, in general, immune reactions to infections differ between men and women. Genetic, hormonal and environmental factors result in stronger innate and adaptive immune responses in women [28]. More specifically, the humoral immune response to malaria parasites is stronger in women than in men [29]. However, women acquiring a malaria infection while pregnant suffer more severe malaria symptoms and complications related to their pregnancy, which are further exacerbated by the sequestration of malaria-infected erythrocytes in the placenta [30]. Secondly, mosquitoes might be differentially attracted to men or women, for a wide range of reasons, e.g. differences in the odours released by the skin microbiome [31–33] and skin emanations [34], or differences in metabolic rate leading to differences in levels of exhaled CO_2_ [35]. Thirdly, human behaviour contributes towards potential exposure to mosquitoes and can be influenced by gender [36]. Current epidemiological models assume random biting behaviour of mosquitoes, although this assumption is thought to be false [37]. In fact, some studies have found differences in observed biting behaviour between medically important mosquitoes. For example, *Anopheles gambiae* showed an age-specific sex difference in biting behaviour [23], and *Aedes aegypti* females exhibited a preference for young adults and males [26], possibly due to their opportunistic host choice. Additionally, one study indicated that the malaria parasites *Plasmodium falciparum* and *Plasmodium vivax* affected adult males more than females in a hypoendemic region where adults were naïve to the infections [38].

The objective of this study was to develop a reliable technique to determine the host sex of a mosquito’s blood meal to provide valuable information at a much lower cost than current STR genotyping kits. Aside from the cost of DNA extractions and general lab consumables, the PowerPlex 21 System (Promega) STR kit costs around £ 19 per run, whereas the cost of the method reported here is around £ 2 per run. One molecular sex marker, amelogenin, is used in commercially available STR kits for forensic investigations [39], and the regions present on both the X and Y chromosomes can be amplified simultaneously [40]. The amelogenin-specific primers used in this study are specific to humans and chimpanzees and amplify a fragment on both the X and Y chromosomes that can be differentiated by size [40]. A BLAST search for targets in all vertebrate genomes confirmed the specificity of the amelogenin-specific primers for human and chimpanzee X and Y chromosomes. Therefore, only blood meals taken on humans (or chimpanzees) should result in a positive amplification and polymerase chain reaction (PCR) products of the right size.

Analysing mosquito blood meals and finding new methods for the rapid and cheap identification of the sex of human hosts from mosquito blood meals could help re-evaluate and optimise disease transmission models. The objective of this study was to develop a relatively cheap and accessible sex determination method for the identification of mosquito blood meals with the aims of (1) correct identification of the sex of the human hosts of blood fed mosquitoes, and (2) proof of concept that the assay is relevant to field studies using wild-caught mosquitoes that have fed up to 48 h before sample collection.

## Methods

### Mosquito rearing

All mosquitoes were maintained at 27 ± 2 °C, 60 ± 10% humidity, 12-h:12-h light:dark photoperiod and fed only on 10% glucose solution before the experiments.

Dried *Ae. aegypti* eggs were imported from Fiocruz, Brazil, in 2016 and have been continuously reared in the laboratory since. Eggs collected and dried on filter paper (Whatman) were hatched in distilled water and fed with dry dog food cubes (Bakers Puppy, Purina). Fat was skimmed when necessary and pupae separated according to age. Adult mosquitoes aged 5–7 days were fed horse blood, and eggs were laid on wet filter paper. Filter papers with eggs were dried and rehydrated as needed.

The colony of *Anopheles coluzzii* has been in culture at the Natural Resources Institute, University of Greenwich, since 2017, and was isolated from a colony established in the same year by the Institut de Recherche en Sciences de la Santé, Burkina Faso. Eggs were hatched in about 1 l of 10% saline water with approximately two grains of baby rice (organic baby rice, Aptamil) and a few flakes of fish food (TetraMin flakes, Tetra), which were continuously supplied until the larvae pupated. Pupae were collected and separated according to age. Adult mosquitoes aged 5-7 days were blood-fed on a human arm, and eggs collected on wet filter paper. Eggs were then transferred to tanks with 10% saline water.

### Sample collection

Mosquitoes aged 5–7 days (*Ae. aegypti*) or 7–12 days (*An. coluzzii*) were allowed to engorge on a volunteer’s arm without interruption. After blood-feeding, mosquitoes were provided with water *ad libitum* in a feeder to reduce mortality. Three mosquitoes were killed immediately (0 h) after feeding by smearing their abdomens onto grade 54 filter paper (Whatman). A further three mosquitoes per volunteer were sampled 6, 12, 24, 36, 48 and 60 h after the blood meal. The Sella blood digestion status (following Detinova [42]) was determined by stereomicroscopy before the mosquito samples were preserved on filter paper and stored at − 20 °C. The blood digestion status scale (following Detinova [42]) was slightly adapted to reflect the subtle differences in colour of the blood, and 0.5 increments were used. The scale ranges from 2 (freshly fed and a fully engorged abdomen with bright red blood) to 7 (no blood visible and eggs fully developed). In this study, a score of 7 was also allocated to mosquitoes without visible ovaries, as it can take more than one blood meal for *An. coluzzii* to develop eggs. A total of 10 volunteer hosts (5 female, 5 male) were recruited, and ethical approval for this was provided by the University of Greenwich’s University Research Ethics Committee (reference number 17.2.5.11).

### DNA extraction and quantification

DNA was extracted from blood-fed *Ae. aegypti* and *An. coluzzii* using the DNAeasy kit (Qiagen, London, UK) following the protocol for purification of total DNA from insects using the DNeasy Blood & Tissue Kit. The final elution was performed twice in the same 50 µl of double-distilled H_2_O to maximise the quantity of DNA retrieved. Positive controls were collected by spotting 5 µl blood taken with a blood lancet from one male and one female volunteer directly onto grade 54 filter paper (Whatman). DNA was extracted following the same protocol, with a final elution of 100 μl. Negative controls were collected from an unused grade 54 filter paper (Whatman), unfed *Ae. aegypti* and *An. coluzzii* legs and abdomens, and DNA was extracted as described above for the mosquito blood meals. DNA was quantified by a NanoDrop 2000 Spectrophotometer (Thermo Scientific).

### Sex-determination PCR protocol

PCR reactions were performed according to the DreamTaq Green PCR Master Mix (2X) (ThermoFisher Scientific) protocol (25 µl reaction volume), with 2 µl (*Ae. aegypti*) or 1 µl (*An. coluzzii*) of extracted DNA and 400 nM forward (CTGATGGTTGGCCTCAAGCCTGTG) and reverse (TAAAGAGATTCATTAACTTGACTG) primers from Nakahori et al. [40]. PCR cycling conditions were as follows: 95 °C for 3 min followed by 40 cycles of 95 °C for 30 s, 56 °C (*Aedes*) or 58 °C (*Anopheles*) for 30 s and 72 °C for 1 min, and a final extension step of 72 °C for 7 min. PCR products were visualised on a 2% agarose gel. Samples that yielded no visible PCR product were subjected to the same PCR protocol once again, with 2 µl and 4 µl samples for *An. coluzzii* and *Ae. aegypti*, respectively.

### Data analysis

R version 3.6.0 was used for all tests [43]. A generalised linear model with binomial errors was used for* y*-success proportion variates and a linear regression model for Sella score* y*-variates.

## Results

### Blood digestion

**Fig. 1:**
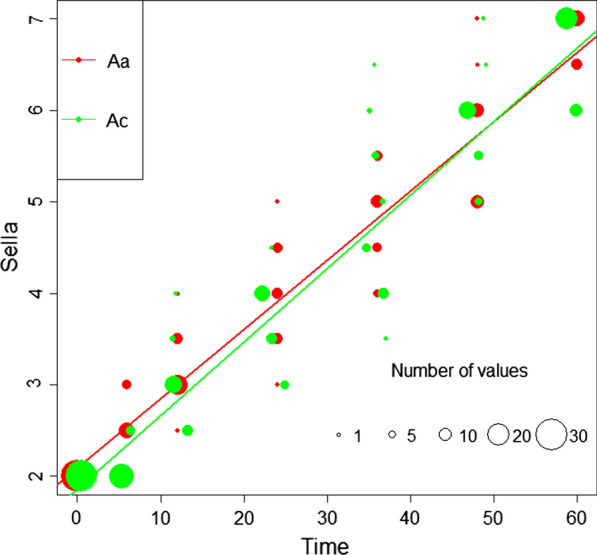
Distribution of Sella scores measured at 0, 6, 12, 24, 36, 48 and 60 h after blood-feeding; *n* (per species and per timepoint) = 30. *Aa* *Aedes aegypti*, *Ac* *Anopheles coluzzii*

Blood meal digestion was assessed using Sella scores, a method adapted from Detinova [42], which cover a range from 2 (fresh blood meal) to 7 (completely digested and no blood visible) [42]. Sella scores for each timepoint were recorded and used to compare blood meal digestion between *An. coluzzii* and *Ae. aegypti* (Fig. 1). Blood digestion scores were highly significantly correlated with time [*F*_(1, 416)_ = 5119.5692, *p* < 0.001, ANOVA] and showed that blood digestion increased linearly with time. Significant differences were detected between the species [*F*_(1, 416)_ = 5.6092, *p* = 0.01832]. Species and time together had a significant effect on the Sella score observed [*F*_(1, 416)_ = 4.6892, *p* = 0.03092].

### Sex determination using PCR

**Fig. 2:**
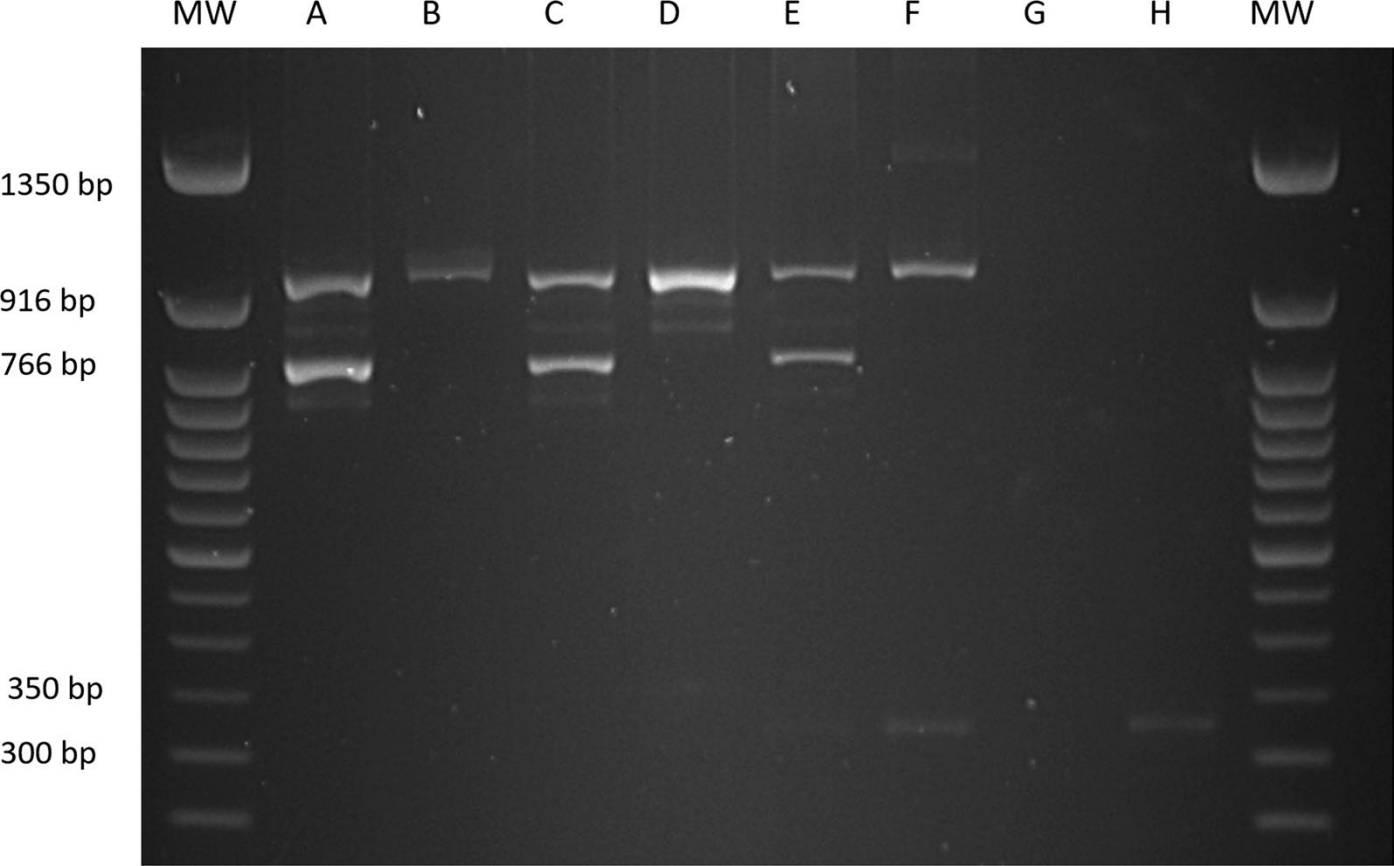
Polymerase chain reaction (PCR) product from DNA extracted from a blood droplet from a male donor (*A*) and a female donor (*B*), from *Ae. aegypti* blood-fed on a male (*C*) and a female (*D*) donor, from *An. coluzzii* blood-fed on a male (*E*) and a female (*F*) donor, from the body of an unfed *Ae. aegypti* (*G*) and an unfed* An. coluzzii* (*H*).* MW* 50-base pair (*bp*) ladder

The primers chosen for the amelogenin locus can amplify *AMELX* (NC_000023.11) and* AMELY* (NC_000024.10) simultaneously. Amplification of the X-chromosomal locus resulted in one band of 977 base pairs (bp) (*AMELX*) and the Y-chromosomal locus generated a band of 790 bp (*AMELY*) (Fig. 2).

The PCR assay was first tested on DNA extracted directly from blood spots and then on blood-fed mosquitoes. Samples from a female volunteer (blood spot and blood meal) were sequenced to confirm the presence of* AMELX* and confirm the amplification of the correct product. Unfed female *An. coluzzii* and *Ae. aegypti* were used as negative controls, and no bands of the size of* AMELX* or* AMELY* were detected. An additional band of around 325 bp was found in samples from *An. coluzzii* blood meals and the negative control of DNA only from an unfed female *An. coluzzii*, confirming this to be amplification of mosquito DNA. However, a BLAST search did not find any binding sites for the primers and sequencing of the product was unsuccessful.

#### Percentage of successful amplifications

**Fig. 3:**
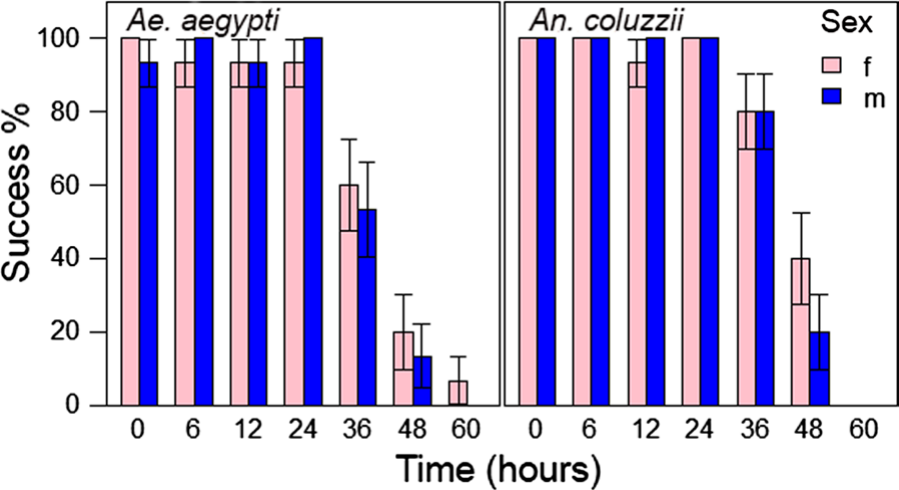
Percentage of successful PCR amplifications for* Ae. aegypti* and *An. coluzzii* fed on female (*f*) and male (*m*) hosts. No significant differences could be found in the efficiency of the PCR product for male or female human DNA (*p* = 0.67).* n* (per bar) = 15.* Error bars* represent the SEs from the general linear model

The PCR assay for* AMEL* was performed on DNA extracted from *An. coluzzii* and *Ae. aegypti* females collected at 0, 6, 12, 24, 36, 48, and 60 h after feeding. Mosquitoes were fed on a total of 10 human volunteers, and 3 individual mosquitoes were collected for each time point and mosquito species.* AMEL* was detected in 93.3–100% of samples up until 24 h post-feeding in both mosquito species (Fig. 3). Success dropped thereafter to 56.6% and 80% at 36 h after feeding for *Ae. aegypti* and *An. coluzzii*, respectively. Forty-eight hours after feeding, a sixth of the *Ae. aegypti* blood meals and 30% of *An. coluzzii* blood meals still yielded a successful amplification. However, 60 h after feeding,* AMEL* could only be successfully amplified in 3.3% of the samples from *Ae. aegypti* blood meals and none of the *An. coluzzii* blood meals.

A three-way analysis of deviance showed that the sex of the host did not significantly influence the success of PCR in either mosquito species (*p* = 0.67286). However, significant differences in the global average for successful PCR amplification were found between the mosquito species (*p* = 0.01039). These differences between the mosquito species were only apparent 36 h after feeding, where 56.6% of *Ae. aegypti* blood meal samples resulted in a successful amplification compared to 80% of *An. coluzzii* blood meal samples. The time that had elapsed after the blood meal was correlated with PCR success: the more time that had elapsed post-feeding, the lower the percentage of successful sex determination (*p* < 0.001).

#### Correlation with the Sella score

**Fig. 4:**
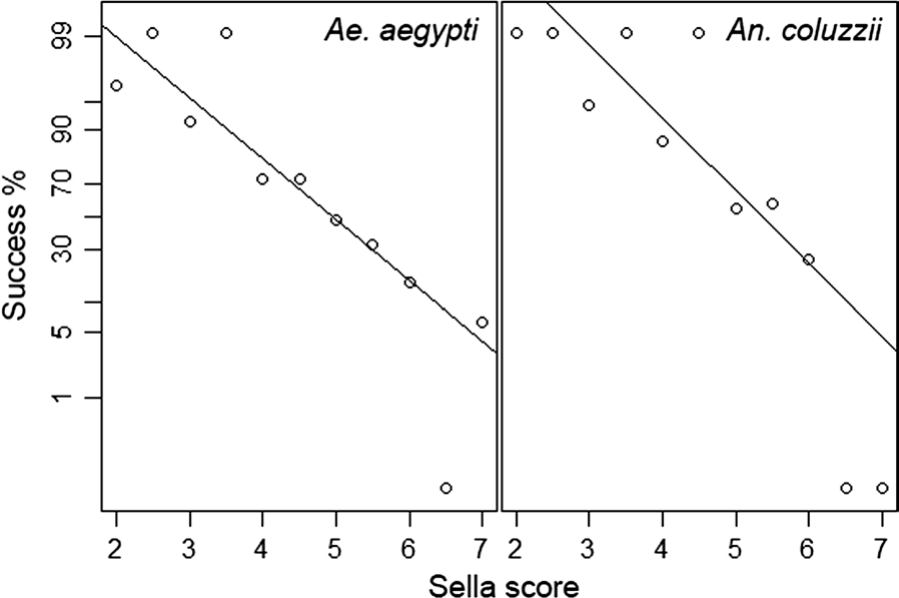
Analysis of covariance using a generalised linear model and a logit link

For the development of a protocol for field-caught mosquito, Sella scores were evaluated as a way of assessing blood digestion and consequently to predict the success rate of the amplification of human DNA. Subtle differences between the species were detected (*p* = 0.03678) in a general linear model analysis of covariance with a difference in the* y*-intercepts of the regression lines. Success of the PCR assay decreased with an increase in the Sella score (Fig. 4). The Sella score was highly significantly correlated with the likelihood of a positive PCR result (*p* < 0.001); the lower the Sella score the higher the percentage of successful PCR amplifications.

## Discussion

A better understanding of the dynamics of host biting can be critical to a better understanding of the spread of vector-borne disease. The method described here is a robust new tool for the analysis of blood meals in two species of anthropophilic mosquitoes. We have demonstrated that blood meal digestion measured by the Sella score is a good tool for prediction of the likelihood of successful amplification, reducing the number of negative PCR runs, and thus saving both effort and cost. Large-scale epidemiological studies of mosquito biting behaviour will benefit from this method for sex determination due to its cost effectiveness and the use of simple equipment that is readily available in most molecular laboratories. Conducting more epidemiological studies that analyse the blood meals of field-caught mosquitoes can help establish how mosquitoes behave naturally, as opposed to under laboratory conditions. For example, when mosquitoes are given the choice between two humans, or are exposed to a range of host odours, the influence of human behaviour on how mosquitoes locate their hosts is not taken into account. The inverse relationship between blood meal digestion and likelihood of successful PCR amplification has been previously investigated. Santos et al. [44] reported a Sella score of 7 for *Ae. aegypti* 54 h after it took a blood meal, and the highest number of successful amplifications at the lowest Sella scores [44], which is on a par with our results that showed the absence of blood after 60 h in the majority of mosquitoes. Similarly, host species identification was highest for *Ae. aegypti* (Sella score 2), with nearly 100% amplification success, dropping to 25% for a Sella score of 5 [45]. However, any method analysing blood meal digestion in laboratory-bred mosquitoes also needs to be tested on field-caught mosquitoes due to the potential influence of environmental [46] and other factors, such as if the blood meal is the mosquito’s first [42]. Nevertheless, the relationship between the Sella score and PCR amplification reported in the present study indicates that the protocol described here is valid for future field studies and can limit the number of PCRs that would likely not yield a result.

Differences in blood meal digestion observed between the mosquito species were particularly pronounced 6 h post-feeding. This could be explained in part by different mechanisms of thermoregulation in these species. For example, prediuresis occurs in *An. coluzzii* immediately after it starts to feed, whereas in *Ae. aegypti* this does not occur [47]. Prediuresis is generally thought to be a means of concentrating erythrocytes, but the urine produced also contains small amounts of ingested blood [47], potentially decreasing the amount of total blood in the abdomen.

A prerequisite for future field studies is the evaluation of the post-feeding interval and its influence on the detection success of a method. In the case of ELISA, human serum can be detected in different *Anopheles *spp. for up to 24 h [48] and in *Ae. aegypti* for up to 39–40 h [49]. The first studies that genotyped mosquito blood meals and matched the profiles to inhabitants of nearby dwellings were conducted in the 1990s. Gokool et al. [50] found that only 35% of blood meals gave profiles, whilst Coulson et al. [51] detected full genetic profiles up to 10-15 h after an *An. gambiae* blood meal; however, a method detecting human blood meals for a longer post-feeding interval would be beneficial for large-scale epidemiological studies. Previous research has shown that *Ae. aegypti* blood meals can be genotyped up to 26 h after feeding [52] and with a 70% success rate after 24 h [53]. The key caveat being that these studies used genetic methods available at the time; the expansion in sensitivity and accuracy of contemporary genetic tools provides opportunities to revisit some of these approaches for the detection of multiple host blood meals and sex determination. A more recent study using a commercial DNA genotyping kit showed a better success rate than these earlier studies, namely successful detection of full 16 loci profiles from all tested mosquitoes up to 32 h post-feeding, irrespective of mosquito genus, and up to 48 h in Culicinae mosquitoes [54]; however, these kits and the equipment required are not readily available.

Variability between studies regarding the likelihood of success of PCR amplifications could be due to the following factors: (1) storage time and temperature of mosquitoes and DNA, (2) DNA extraction method, and (3) mosquito species. In our study, mosquitoes were squashed on filter paper, a method found to be particularly advantageous for future PCR success [55], and useful for transport and storage of field-caught mosquitoes [56]. The DNA extraction method can have a significant effect on the quality of the DNA, e.g. Martinez-de la Puente et al. [57] found that the Qiagen blood and tissue kit improved amplification success compared to the cheaper HotSHOT technique. Previous studies have also examined methods such as phenol/chloroform DNA isolation [26, 53], which yield less host DNA and result in a lower amplification success rate.

In the present study, human DNA was detectable for longer in *An. coluzzii* than in *Ae. aegypti*. However, *Ae. aegypti* has been reported to take up to a mean of 5 µl of blood when feeding on a restrained host [58], while *An. coluzzii* consistently imbibed less than 5 µl of blood [59]. Additionally, Curic et al. [54] compared the amount of human DNA in Culicinae and Anophelinae blood meals and found greater amounts in Culicinae; however, a different anopheline species was used than in the present study, which could account for the differences between Curic et al.’s [54] and our results. Mukabana et al. [60] found that amplification success was not affected by blood meal size but only by digestion; however, only blood meals of different *Anopheles gambiae s.s.* specimens, not different mosquito species, were analysed [60]. The variation in success of PCR amplifications can be explained by species-specific alterations in the speed of digestion. The method of collection of mosquito blood meals may lead to the presence of mosquito DNA, providing off-target priming sites for human amelogenin PCR primers. A Blast search confirmed no matches of the primers used here in any mosquito genomes; however, the PCR assay on DNA samples from fed and unfed *An. coluzzii* body and legs resulted in an additional band of approximately 300 bp. The molecular determination of mosquito species from mosquito DNA enables species-specific differences to be observed in the field.

In future epidemiological studies, the method described here can supplement other methods such as PCR-based species identification (see e.g. [18, 61, 62]) without the need for reference samples from the inhabitants of an area. Furthermore, DNA-based methods can be used to increase the epidemiological value of such studies, including methods to estimate age of the blood host, e.g. DNA methylation [63] or signal joint T-cell receptor excision circle quantification [64]. One drawback of this method is the potential for misidentification of multiple blood meals. However, using the Agilent Bioanalyzer [65] or MALDI-ToF [66] for genotyping could provide a cost-effective alternative to capillary electrophoresis-based systems.

## Conclusions

The method described here should be helpful in field studies that aim to broaden our understanding of how mosquito species, including those that exhibit outdoor feeding, find their hosts, and ultimately inform disease-transmission models of vector-borne diseases. This should open the door to a better understanding of how gender-based behavioural patterns influence encounters between humans and blood-feeding vectors of disease.

## Data Availability

The datasets used and analysed during the current study are available from the corresponding author on reasonable request.
